# Performance Based Financing and Uptake of Maternal and Child Health Services in Yobe Sate, Northern Nigeria

**DOI:** 10.5539/gjhs.v5n3p34

**Published:** 2013-01-29

**Authors:** Garba M. Ashir, Henry V. Doctor, Godwin Y. Afenyadu

**Affiliations:** 1 Operations Research Unit, Partnership for Reviving Routine Immunization in Northern Nigeria; Maternal, Newborn, and Child Health (PRRINN-MNCH) Programme, Damaturu, Yobe State, Nigeria; 2 Department of Paediatrics, University of Maiduguri, Maiduguri, Nigeria; 3 Columbia University, Mailman School of Public Health, Department of Population and Family Health, New York, USA; 4 Operations Research Unit, PRRINN-MNCH Programme, Abia State House, Abuja, Nigeria; 5 Health Systems Research Manager, PRRINN-MNCH Programme, Kano, Nigeria

**Keywords:** health systems research, maternal and child health, maternal mortality, performance based financing

## Abstract

Reported maternal and child health (MCH) outcomes in Nigeria are amongst the worst in the world, with Nigeria second only to India in the number of maternal deaths. At the national level, maternal mortality ratios (MMRs) are estimated at 630 deaths per 100,000 live births (LBs) but vary from as low as 370 deaths per 100,000 LBs in the southern states to over 1,000 deaths per 100,000 LBs in the northern states. We report findings from a performance based financing (PBF) pilot study in Yobe State, northern Nigeria aimed at improving MCH outcomes as part of efforts to find strategies aimed at accelerating attainment of Millennium Development Goals for MCH. Results show that the demand-side PBF led to increased utilization of key MCH services (antenatal care and skilled delivery) but had no significant effect on completion of child immunization using measles as a proxy indicator. We discuss these results within the context of PBF schemes and the need for a careful consideration of all the critical processes and risks associated with demand-side PBF schemes in improving MCH outcomes in the study area and similar settings.

## 1. Introduction

Reported maternal and child health outcomes (MCH) in Nigeria are amongst the worst in the world, with Nigeria second only to India in the number of maternal deaths (UNICEF, 2009; WHO, 2011). At the national level, maternal mortality ratios (MMRs) are estimated at 630 deaths per 100,000 live births (LBs) but vary from as low as 370 per 100,000 LBs in the southern states to over 1,000 deaths per 100,000 LBs in the northern states. For example, MMRs in the North East and North West zones of Nigeria are estimated at 1,549 and 1,049 deaths per 100,000 LBs, respectively (Doctor et al., 2012; Federal Ministry of Health of Nigeria et al., 2009). Between 2008 and 2011, Nigeria’s under-five mortality rate ranged between 157 and 172 deaths per 1,000 LBs (National Bureau of Statistics, 2012; National Population Commission and ICF Macro, 2009). Recent estimates also show that Nigeria has achieved only an average of 2.0% reduction in under-five mortality per year between 1990 and 2010 and it needs to achieve an annual reduction rate of about 10% between 2010 and 2015 to meet Millennium Development Goal (MDG) 4 of reducing under-five mortality by two-thirds between 1990 and 2015 (Murray et al., 2007).

Achieving MDGs 4 and 5 (reduction of maternal mortality by 75% between 1990 and 2015) in northern Nigeria cannot therefore be a matter of ‘business as usual’. Innovative ways must be identified to fast track the coverage and uptake of quality maternal, newborn, and child health services. One of such innovations is performance based financing (PBF; also known as reward schemes or conditional cash/incentive transfer). PBF schemes have yielded some positive results in some deprived and challenging operational settings which have weak health systems such as Afghanistan and in post war ravaged Rwanda (Honda, 2012; Meessen et al., 2011; Meessen et al., 2006). PBF schemes have also shown to increase skilled delivery in India, increased the outputs of non-governmental organizations in health in Haiti, and increased immunization coverage (Eichler et al., 2007). These schemes are designed to provide cash and goods as incentives or rewards against measurable actions or the achievement of pre-defined performance targets. They are targeted at solving a predetermined performance problem including appropriate health behavior. On the one hand, the incentives may therefore target performance problems relating to the service provider’s behavior that could be defined as the behavior of the individual health worker, the facility, a local government or a ward health team. On the other hand, the incentives may also target the health behavior of communities, households, or the individual. Thus, a PBF scheme may target performance problems on both the supply and the demand side of the health systems. In Nigeria, most of the maternal, new born, and child deaths occur in the northern states, a region that is challenged by weak health systems, poverty, violence, and some socio cultural practices that are detrimental to the health of women and children.

The Partnership for Reviving Routine Immunization in Northern Nigeria; Maternal, Newborn, and Child Health (PRRINN-MNCH) Programme (hereafter “the program”) was established by a consortium led by Health Partners International (United Kingdom (UK)), Save the Children UK (now Save the Children International), and GRID Consulting (Nigeria), with the Nigerian State Ministries of Health and local officials of Jigawa, Katsina, Yobe, and Zamfara States of northern Nigeria. The program promotes routine immunizations through revitalization of primary health care services and focuses on women’s basic education related to the importance of utilizing health facilities for women’s and children’s health, increasing demand for MNCH services, ensuring that all women know maternal danger signs and are assisted by skilled staff during delivery as well as have access to emergency care. The Department for International Development (UK) and the Norwegian Government fund the program.

As part of its activities, the program commenced a pilot PBF in Yobe State in 2011, aimed at increasing coverage and uptake of MNCH services. This paper presents preliminary results on some of the processes and outcomes of an operations research (OR) pilot study funded by the program on the role of demand-side PBF scheme in increasing utilization of MNCH services in Yobe State, northwest Nigeria.

## 2. Methods

### 2.1 Study Location

Based on the 2006 population census of Nigeria, the population of Yobe State was 2.3 million people with the dominant ethnic groups of Fulani, Hausa, and Kanuri. Islam is also the predominant religion. Agriculture and fishing are the main sources of income. The public health system in Yobe State consists of five operational clusters, each targeting 500,000 people as proposed by World Health Organization. Based on this system, the primary health clinic (commonly referred to as ‘24/7’) is first point of contact with the health system run by a community health extension worker (CHEW). From the ‘24/7’, referrals can be made as needed to Basic Emergency Obstetric Care (BEOC) and to the Comprehensive Emergency Obstetric Care (CEOC). All the public health facilities receive free drugs and medical supplies, staff salaries, and a budget for running costs from the government. In this cluster, there is no private health centre but there are numerous and often unregulated drug vendors practices.

As part of the program’s activities in Yobe, Geidam Local Government Area (LGA) was selected as the learning LGA for OR studies. Geidam is located in the semi-arid northern part of Yobe where most people live in sparsely distributed rural and hard-to-reach settlements. Out of the 11 intervention areas in Geidam LGA, six intervention areas were randomly selected for the PBF pilot study namely, Danburam, Gallaba, Kelluri, Mala-Ngobtor, Moborti, and Shuwari. Gumzigi and Kayyiya were selected as comparison areas. All settings were located within five Kilometers from the health facilities with well designed MNCH integrated outreach service by the CHEWs. All communities have existing community engagement structures, community volunteers (CV), community savings (CS), trained emergency transport scheme (ETS) driver, and blood donors (BD). Before its implementation, the health research and ethics committee of Yobe State Ministry of Health granted ethical approval for the pilot study.

### 2.2 Study Population

The total population of the PBF intervention communities was projected to be 30,000 according to the 2006 population census. Twenty-two percent of the total population were women of childbearing age, with an estimated 1,500 pregnant women at any given time. The OR baseline survey conducted in 2009 showed that about 19% (6,000) of population were under-five years and would therefore need immunization services. This meant that in any given year, the target population for the PBF incentive was 7,500 (women and children). In addition, the program worked with six village development committee (VDC) and maternity staff of Kelluri BEOC and Geidam General Hospital (CEOC), responsible for referral emergency obstetric services at their facilities. In the communities with PBF, the scheme is also working with six CHEWs, 150 CVs, and 11 CS groups.

### 2.3 Study Design

The study design was a community-based quasi experimental prospective study. A two-cell study design was chosen as follows: Cell A comprised of communities with PBF disbursement through VDC; Cell B consisted of communities without PBF. The selection of intervention communities was based on the following criteria: (1) communities in the Learning LGA as intervention area; (2) communities where community engagement activities had been established, existence of CVs, CSs, ETS drivers, and BD group.

For the purpose of the pilot, an intervention community was defined as a community that fulfilled the aforementioned criteria plus PBF whereas the control communities were those which did not meet both criteria (cell B). The pilot study design is summarised in Table 1.

**Table 1:** Pilot study design of ongoing (Jan-Dec 2012) Yobe State PBF

Cell A: Communities with PBF (cash)	Cell B: No PBF support
*Characteristics of intervention area*	*Characteristics of control area*
• Geidam LGA	• Geidam LGA
• Vouchers disbursement through VDC (6 communities)	• No incentive (2 communities)
• Facilities: MCH, Kelluri, and GH Geidam	• Facility: MCH Gumsa

The EOC facilities were selected in order to provide EOC services and were also dichotomized into intervention and control facilities. Kelluri (BEOC) and Geidam GH (CEOC) are the intervention facilities whereas Gumsa MCH facility (BEOC) is the control facility. The facilities are selected with reference to the category of communities they are serving. To ensure sufficient quality and safe delivery services, all the selected facilities (1) can provide the minimum package of services as recommended by the State Ministry of Health for a health centre, (2) have at least one skilled midwife available in time of need, and (3) have relatively high utilization record for antenatal care (ANC) and delivery.

The management of the PBF scheme was assigned to the incentive management committee. A Fund Holder Committee (FHC) was established to support and supervise the activities of the voucher management agency (VMA) and monitoring and evaluation committee. All community members have names and contact phone numbers/addresses of all the VMA members in the areas who can be contacted when clients need their services. Performance indicators were defined as receipt of 1 and 4 ANC visits; skilled delivery for women; and DPT3 and measles immunization for children. Any woman/child that achieves such performance at any of the designated MCH facilities qualifies for the cash voucher. The cash amount is N500 (Nigerian Naira) per target except for skilled birth delivery which is N1000 (approximately US$3 and US$6, respectively, at the time of this study). The cash incentive was arrived at after consideration of a number of factors such as the approximate cost of transport and feeding. In each area, the management of the voucher was sub-contracted to the village health committee to operate as a VMA. The voucher recipients were pregnant women and under-five children in their respective catchment area. Under exceptional circumstances (e.g., the pregnant woman being sick or unavailable), close relatives received the vouchers on behalf of the pregnant women.

Among other things, staffs in the intervention facilities were retrained to provide maternal services in a timely and professional way. An incentive was also given to the facilities as a strategy to address the poor health care providers’ attitude, a critical factor associated with underutilization of health facilities and driven by findings from community consultation conducted in the study areas. With this arrangement, intervention facilities receive social incentives on a quarterly basis as agreed during stakeholders meetings. Each client was given a cash voucher with three detachable coupons which can be redeemed from the disbursement agency. The coupons are signed and stamped at the MCH centre. One of the coupons is kept at the MCH facility. Women are expected to submit one coupon to the VMA in exchange for the cash incentive. The woman keeps the last stamped coupon. At the end of each month, the FHC pays the village health committee on the basis of the number of signed coupons. The FHC provides cash advances for payment of clients. A series of monitoring mechanisms have been put in place and are led by supervisors of the FHC, the OR team, and the monitoring and evaluation assistant. Monitoring and evaluation mechanisms were developed by refining the existing health management information system data capture tools to capture utilization of the targeted service. In addition, the research team set up validation and accountability tools at the central level using harmonized quantity verification tools (for data quality) and developed quality evaluation checklists (for service quality).

### 2.4 Data Collection

Process and outcome data were collected at the initial stage and throughout the pilot phase. Data are collected monthly followed by monthly review meetings for information sharing and explanation of the technical developments surrounding the PBF. All data collection forms were reviewed for completeness, accuracy, and reliability. Such information is fed back into strengthening the implementation process.

### 2.5 Outcome Measures and Statistical Analysis

Determination of each performance indicator at every visit was ascertained from the client ANC and immunization records as well as from the facility-based record. All participating families are expected to produce their ANC and child’s immunization card at every visit. Where women failed to produce the cards for any reason such as loss of the cards, the clinic-based ANC and immunization records were used for data collection (with the facility subsequently generating a “new” card). Data were entered into an Excel spread sheet and subsequently analyzed with STATA version 11 (StataCorp, 2009). Basic descriptive statistics as well as tests of significance for proportions were computed.

## 3. Results

### 3.1 General Characteristics of the Study Population

We present results of the PBF pilot study in Yobe State for women with four ANC visits, skilled delivery, and measles immunization coverage for children. Results are presented for each month of the pilot study from November 2011 to April 2012. Based on the description of the “study population” presented in Section 2.2, Table 2 presents a summary of the target population for the intervention and control areas and for the selected indicators. Overall, results show that the population of the catchment control area was more than the population of the intervention area. Specifically, the catchment population in the control area was 1.78 times more than the catchment population in the intervention area for the expected pregnant women and children under one year. The absolute differences in the three indicators of ANC, delivery, and measles immunization between the intervention and control areas are statistically significant (chi-square 106.58; p=0.000). The results do not show any consistent trend from November 2011 to April 2012.

**Table 2:** Target population for the study area and the selected PBF pilot study indicators, Nov 2011-April 2012

Characteristics	Nov	Dec	Jan	Feb	Mar	Apr
*Intervention area*						
Women with 4 antenatal care visits	45	27	31	13	47	29
Women with skilled assistance at delivery	15	11	14	7	8	8
Children receiving measles immunization	109	72	82	61	56	75
*Control area*						
Women with 4 antenatal care visits	8	4	4	12	21	10
Women with skilled assistance at delivery	0	0	3	6	4	7
Children receiving measles immunization	78	44	23	11	72	23

Catchment area population	21,520 (38,215)*					
Expected pregnant women (5% of population)	1,076 (1,911)*					
Expected pregnant women per month	90 (159)*					
Expected under-1 year children (4% of population)	861 (1,529)*					

Notes:

* The numbers in parentheses are for the control area whereas the others are for the intervention area. Differences in ANC, skilled assistance at delivery, and measles immunization between the intervention and control areas are statistically significant (chi-square = 106.58; p=0.000)

### 3.2 Antenatal Care Attendance

**Figure 1:**
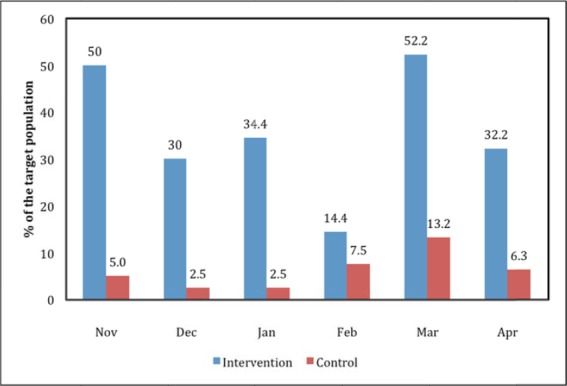
Comparison of fourth ANC visit by study area, Nov 2011-Apr 2012, Yobe State, Nigeria

The specific indicator of interest was women with four ANC visits. The results from Table 2 were converted into rates based on the population at risk (Figure 1). Results show that half of the target women in the intervention area had four ANC visits in November 2011 compared with 5% of the target women in the control area. In December 2011, women with four ANC visits accounted for 30% of the target population compared with 2.5% in the control area. Although the magnitude consistently varies, generally and for all the months between November and April, the intervention area consistently registered a higher proportion of women with four ANC visits than their counterparts. Differences between intervention and control areas for the ANC visits were statistically significant (chi-square=12.82; p=0.025).

### 3.3 Assistance at Delivery

**Figure 2:**
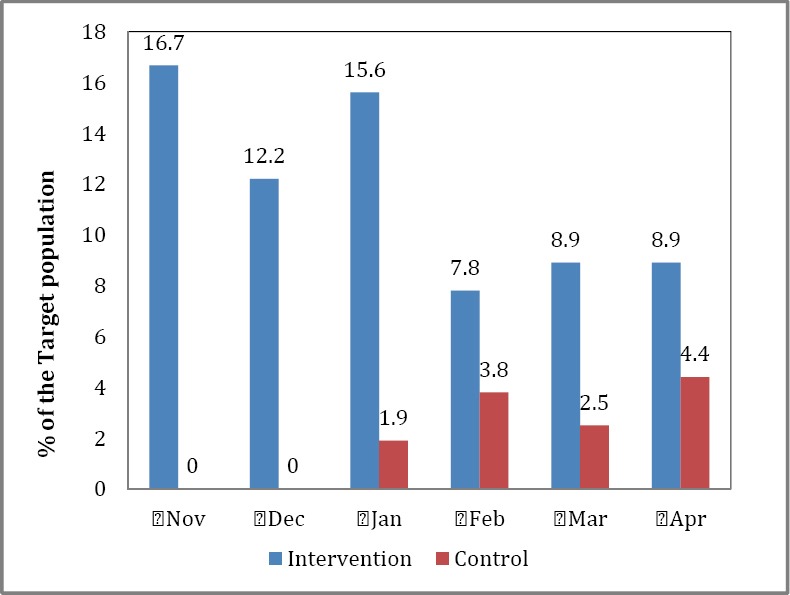
Women with skilled assistance at delivery by study area, Nov 2011-Apr 2012, Yobe State, Nigeria

Results displayed in Figure 2 show that in November 2011 none of the target women in the control area had skilled assistance at delivery compared with 16.7% of the target women in the intervention area. Similarly, no women in the control area received skilled assistance at delivery in December compared with 12.2% of the target women in the intervention area. The trend changed from January 2012 with women in the control area benefiting from skilled assistance though at a much lower rate than their counterparts (e.g., 1.9% vs 15.6% of target women in the control and intervention area, respectively). Between February and April 2012, the percent of target women in the control area receiving skilled assistance at delivery ranged between 2.5% and 4.4% whereas in the intervention area this ranged between 7.8% and 8.9%. Differences between intervention and control areas for the skilled assistance were statistically significant (chi-square = 12.98; p=0.024).

### 3.4 Measles Immunization

**Figure 3:**
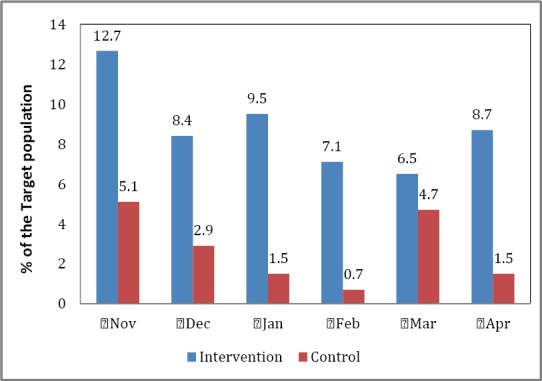
Measles immunization coverage by study area, Nov 2011-Apr 2012, Yobe State, Nigeria

While the percent of the target children receiving measles immunization for children was higher in the intervention area than the control area (e.g., November figures showing 12.7% vs 5.1%, respectively), the differences (Figure 3) between the study area for the period under study were not statistically significant (chi-square = 3.27; p=0.659). These differences, as will be discussed later, are generally related to vaccine stock outs and the fact that Yobe State is far from the national office (Abuja) - about 713 kilometers or 443 miles apart - which controls the distribution of immunization antigens. That is, by virtue of its location it is likely to be disadvantaged in receipt of antigens.

## 4. Discussion

While PBF schemes are swiftly becoming a mainstay of social protection and government welfare programs in Nigeria, there is little evidence about how these programs have affected MCH and what design components in PBFs may lead to favorable outcomes. In this study, process and outcome indicators were documented and our estimates suggested that the demand-side PBF led to increased utilization of key MCH services (ANC and skilled delivery) but had no significant effect on completion of child immunization using measles as a proxy indicator.

The documented effect of PBF scheme on prenatal care can be explained by the financial incentives. The payment of completion of at least four ANC visits is US$3. Women in this region tend to start ANC late in pregnancy (in most cases during the third trimester) and have only the first visit. Therefore, for half of the clients in the intervention area to achieve four ANC visits compared with 5% of women in the control, they have to start ANC much earlier in the pregnancy and attend more frequently. Worthy noting is the fact that health system reforms and the existence of community engagement structures (which mobilizes and educates community members on the benefits of health care utilization) are homogeneous in both the intervention and the control areas. The presence of a comparison group in our study design suggests that the incentive scheme largely explains the increased uptake in MCH services.

Similarly, a significant increase in skill birth delivery was recorded in this pilot. Although majority of women still deliver at home in the study areas (Findley et al., 2012), when compared with baseline finding of 2.9% in both areas, an average increase of 10% suggests that more institutional deliveries occurred among most vulnerable groups. Demand-side financing programs have received criticism for not being able to reach the most disadvantaged women. In a study from Nepal some obstacles in reaching poor women were observed (Barker et al., 2012). The program did not specifically target the poor, thus the distribution of the incentives was skewed towards the wealthier families that were more likely to have an institutional delivery. Our study specifically targeted disadvantaged communities with families having similar socio-demographic background in terms of age, poverty level, education, parity, ANC, and skilled delivery. In this study, the majority of women knew of the program through the existing community engagement structures (CV, CS, ETS, BD, and VDC), hence mobilizing the disadvantage groups from the communities.

Attaining full immunization for a child demands a lot from women: many visits to the clinic and going through complex administrative procedures. The absence of a significant effect of the PBF scheme on child vaccination may be explained by factors beyond the control of clients such as vaccine stock out during the pilot. Further, the selection of measles vaccination as a proxy indicator for fully immunized children contributes to this lack of effect. The payment rate for child vaccination indicators (DPT3 and measles) is $US3 only per indicator, other visits in between were not incentivized. Furthermore, previous studies have found a high drop-out rate of DPT3 in this region (Babalola, 2009). However, despite this lack of significant effect, the childhood vaccination performance is higher in the intervention areas when compared to the do nothing areas. One possible explanation for this finding is that the increased monitoring and supervision led to increased mobilization from the intervention communities by the CVs, among others. Other potential confounders were the general health sector reforms and interventions by both government and donor agencies. However, our findings may not have been biased a great deal by these interventions since they were implemented statewide.

## 5. Conclusion

Although PBF is not a magic bullet for solving all health systems related problems (Ireland et al., 2011), our findings demonstrate that PBF can increase the utilization of MCH services and thus accelerate progress towards the MDGs for MCH. The pilot also shed light on design issues with emphasis on the necessary structures and conditions needed for expansion and replication of this scheme such as the use of VDCs. Other lessons emerged from this pilot include identification of indicators that require many visits to be achieved, the need for incentive packages or payments per visit (which can be sustained), as well as strengthening monitoring and supervision. Nevertheless, being a quasi-experimental study, some bias cannot be avoided. However, initial OR baseline study found that the intervention and control areas are homogenous for most socio-demographic variables thereby minimizing the chance of introducing bias associated with heterogeneous populations. While the analysis is limited to primary data, a population based study using both qualitative and quantitative data would shed more light on health outcomes, quality of care, and health providers’ views. We hope that findings from this study will provide opportunities for policy makers from similar settings to deploy similar PBF schemes in order to improve MCH outcomes. The need for a careful consideration of all the critical processes and risks associated with demand-side PBF schemes in improving MCH outcomes cannot be overemphasized.
